# Carcinogen metabolism, cigarette smoking, and breast cancer risk: a Bayes model averaging approach

**DOI:** 10.1186/1742-5573-7-10

**Published:** 2010-11-16

**Authors:** Nadine Stephenson, Lars Beckmann, Jenny Chang-Claude

**Affiliations:** 1 Division of Cancer Epidemiology, German Cancer Research Center DKFZ, Im Neuenheimer Feld 581, 69120 Heidelberg, Germany

## Abstract

**Background:**

Standard logistic regression with or without stepwise selection has the disadvantage of not incorporating model uncertainty and the dependency of estimates on the underlying model into the final inference. We explore the use of a Bayes Model Averaging approach as an alternative to analyze the influence of genetic variants, environmental effects and their interactions on disease.

**Methods:**

Logistic regression with and without stepwise selection and Bayes Model Averaging were applied to a population-based case-control study exploring the association of genetic variants in tobacco smoke-related carcinogen pathways with breast cancer.

**Results:**

Both regression and Bayes Model Averaging highlighted a significant effect of NAT1*10 on breast cancer, while regression analysis also suggested a significant effect for packyears and for the interaction of packyears and *NAT2*.

**Conclusions:**

Bayes Model Averaging allows incorporation of model uncertainty, helps reduce dimensionality and avoids the problem of multiple comparisons. It can be used to incorporate biological information, such as pathway data, into the analysis. As with all Bayesian analysis methods, careful consideration must be given to prior specification.

## Background

Logistic regression and regression with stepwise selection are standard approaches to assess individual and joint effects of genetic and environmental factors on disease risk. However, one drawback is that the resulting estimates depend on the choice of the underlying causal model, and that hence a different set of covariates may lead to different effect estimates and potentially a different pattern of significance. Moreover, standard regression approaches do not incorporate the uncertainty about our choice of the assumed causal model into the final inference.

An alternative approach to analyze such data in combination is Bayes Model Averaging (BMA) [1], which explicitly accounts for uncertainty with respect to the causal model. BMA specifies prior distributions for model parameters and uses Markov Chain Monte Carlo (MCMC) methods to infer posterior estimates from the priors and from the data. Its inherent model selection feature evaluates different submodels and inference is obtained by averaging over all models considered. By selecting and evaluating a range of submodels, BMA provides a means to reduce dimensionality in the presence of many predictors, when including all variables and their pairwise or higher-order interactions into a logistic model might lead to unstable estimates and bias due to sparse data and correlation [2]. Model selection methods like stepwise regression achieve a similar goal, but do so in a mechanical way, often leading to globally suboptimal and unstable estimates.

**Figure 1:**
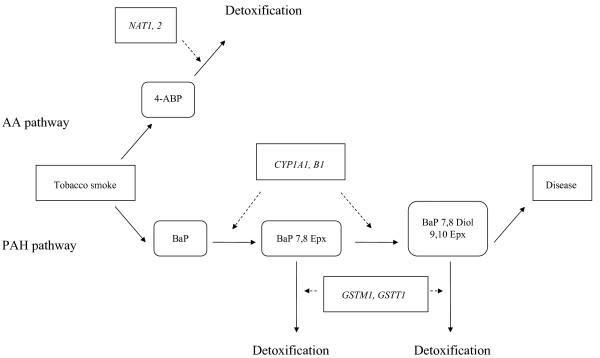
**Directed Acyclic Graph of the proposed pathways**. Measured quantities, i.e. smoking, disease status and genotypes are represented by angular boxes, intermediate metabolites by rounded boxes. Solid arrows mark the metabolic pathways whereas broken arrows indicate the influence of the polymorphisms.

We applied both BMA and logistic regression with and without stepwise selection to data from a case-control study exploring the association of genetic variants in the cigarette smoke carcinogen metabolism and breast cancer. Cigarette smoke is known to contain aromatic amines and polycyclic aromatic hydrocarbons, whose conversion to reactive metabolites by catalyzing enzymes can lead to DNA damage as a first step in breast carcinogenesis. The present population-based case-control study of breast cancer in Germany evaluated the role of genetic polymorphisms in Phase I and II enzymes *NAT1 *and *NAT2 *in the AA pathway and *CYP1A1, CYP1B1, GSTM1 *and *GSTT1 *in the PAH pathway and cigarette smoke exposure in breast carcinogenesis. We analyzed pairwise interactions of polymorphisms as well as interactions of smoking and the polymorphisms to determine an effect on breast cancer risk. The postulated pathways are depicted as a directed acyclic graph in Figure 1.

## Methods

### Data

Data were derived from a population-based matched case-control study on breast cancer conducted in the study regions "Rhein-Neckar-Odenwald" and "Freiburg" of Southern Germany between 1992 and 1995, as described previously [3,4]. Cases were diagnosed by age 50 with invasive or in-situ breast cancer, and two controls were matched to cases by age at diagnosis and study region. Participants completed a self-administered questionnaire assessing demographic factors, anthropometric measures and other known or putative risk factors, including smoking history. All study participants gave written informed consent, and the study was reviewed and approved by the ethics committee of the University of Heidelberg, Heidelberg, Germany.

Smoking behavior was assessed over a lifetime, accounting up to eight different phases of active smoking habits. Cumulative cigarette smoking was quantified in packyears, defined as the number of packs of cigarettes smoked per day multiplied by the number of years the individual has smoked.

For the present study we analyzed polymorphisms in the genes *NAT1*, *NAT2*, *CYP1A1*, *CYP1B1*, as well as the *GSTM1 *and *GSTT1 *deletion polymorphisms. Specifically, *NAT1 *and *CYP1B1 *genotypes were coded as the number of NAT1*10 and CYP1B1*3 alleles, respectively. *NAT2 *was coded rapid acetylating conditional on the presence of at least one NAT2*4 allele, which is characterized by the absence of four point mutations (as previously described in [3]), and slow acetylating otherwise. *CYP1A1 *was either homozygote for the wild-type allele CYP1A1*1, defined by the absence of three point mutations (rs1056827, rs1056836, rs1800440), or otherwise. *GSTM1 *and *GSTT1 *were characterized by the absence of their gene product.

Analyses were adjusted for age, family history of breast cancer in terms of number of affected first-degree relatives, and menopausal status classified as either pre- or postmenopausal, or unknown for women with previous hysterectomy not accompanied by bilateral oophorectomy. Menopausal status was assigned according to the reported state a year before the reference date. Study region showed no effect in an earlier analysis [5] and was hence not considered in the model.

Non-missing genetic and epidemiologic data were available for a total of 654 cases and 1085 controls. A description of the study population and of the genetic variables is given in Table 1.

**Table 1:** Study characteristics of the breast cancer case-control study in Germany and variable definition.

Variable	Definition	Levels	Cases	Controls
			654	1085
Age			42.5 +/- 5.7	42.6 +/- 5.7
				
Family history	first-degree relatives with breast cancer	none	87.80%	94.80%
		at least 1	12.20%	5.20%
				
Menopausal status^a^		premenopausal	78.70%	80.60%
		postmenopausal	6.30%	6.50%
		unknown	15.00%	12.90%
				
Smoking	packyears over a lifetime		8.27 +/- 12.20	6.96 +/- 10.88
				
*NAT1*	number *10 alleles	0	64.10%	68.60%
		1	32.30%	28.60%
		2	3.70%	2.90%
				
*CYP1B1*	number *3 alleles	0	31.00%	28.90%
		1	51.70%	50.90%
		2	17.30%	20.20%
				
*NAT2*	presence of at least one *4 allele	fast acetylator	43.70%	39.80%
		slow acetylator	56.30%	60.20%
				
*CYP1A1*	homozygote for *1 allele	Yes	75.80%	75.10%
		No	24.20%	24.90%
				
*GSTT1*	absence of gene product	No	84.30%	81.90%
		Yes	15.70%	18.10%
				
*GSTM1*	absence of gene product	No	45.70%	48.80%
		Yes	54.30%	51.20%

^a ^Women with a hysterectomy not accompanied by bilateral oophorectomy were classified as unknown

### Statistical Methods

#### General remarks

Interactions were only considered if all constituting main effects were in the model. We further restricted the domain of possible gene-gene interactions to polymorphisms in the same pathway. All variables were treated as continuous and were centered on 0.

#### Logistic regression and backward selection

All logistic regression models contained terms for age, family history, and menopausal status. We tested (i) main effects of smoking and the six polymorphisms separately, (ii) interaction of smoking with each polymorphism, and (iii) gene-gene interactions for polymorphisms in the same pathway. Stepwise regression with backwards selection was used to identify subsets of variables that best explained the data according to the Akaike Information Criterion [6]. We applied stepwise regression to (i) the model containing smoking and all six polymorphisms, and (ii) the model containing all main effects as well as interactions of smoking with all polymorphisms and all gene-gene interactions within pathways.

#### Bayes Model Averaging (BMA)

**Figure 2:**
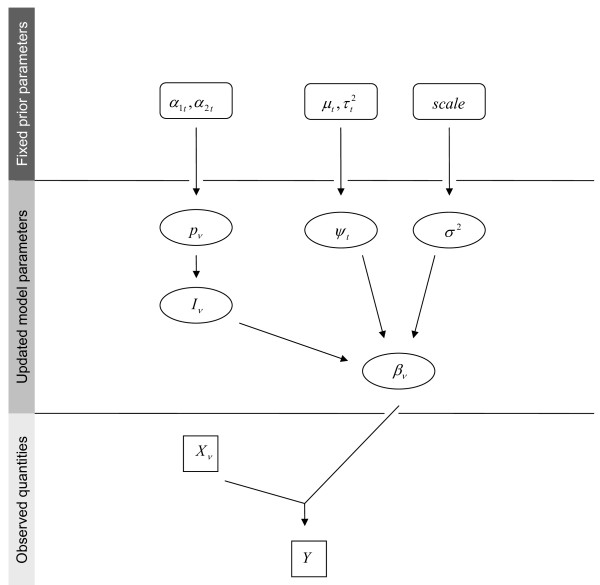
**Directed Acyclic Graph for BMA and its parameters**. Boxes represent observed quantities, ovals parameters to be updated over the course of MCMC, and rounded boxes fixed meta-parameters. Y denotes the dependent variable, and *ν *indexes the sets X of independent predictor variables and *β *of corresponding estimates. I indicates inclusion of the *ν*th variable and is Bernoulli-distributed with parameter p_*ν*_, which, in turn, follows a beta distribution with parameters (a_t_, b_t_) depending on the interaction level t of the variable. The variance of the coefficients *β_ν _*is modeled by a residual variance term *σ*^2 ^following a half-Cauchy prior, and a variance inflation factor *ψ*_t _depending on the interaction level and following a log-normal distribution with mean *μ*_t _and variance *τ*_t_.

The Bayesian model we used is illustrated as a directed acyclic graph in Figure 2. At each iteration step, we considered a logistic model of the form

$$\text{logit}\left(\mathrm{Pr}(Y=1)|X\right)=\sum_{c}\beta_{c}X_{c}+\sum_{\nu }I_{\nu }\beta_{\nu }X_{\nu },$$

where {*X_c_*} consisted of the terms for age, family history and menopause that were included in each model.{*X_ν_*} contained the terms for smoking, the six polymorphisms, as well as interactions of smoking with all polymorphisms, and all gene-gene interactions within pathways. Following Conti et al. [1], *I_ν _*was a binary indicator marking the presence of term *X_ν _*in the model. Assuming that *X_ν _*= *X_rs _*was an interaction term with constituting main effects *X_r _*and *X_s_*, then *I_rs _*= 0 if any of *I_r _*or *I_s _*were 0, formalizing the requirement that all main effects had to be in the model for an interaction to be present.

The following prior distributions were specified for the model parameters. The probability *p_ν _*= Pr(*I_ν _*= 1) was beta-distributed with parameters $\left(\alpha_{1_{main}},\;\alpha_{2_{main}}\right)=\left(1,\;3\right)$ if *X_ν _*was a main effect. This prior corresponded to a marginal inclusion probability of 0.25 for main effects and was chosen to reflect our prior emphasis on models with fewer main effects. Moreover, since the inclusion of interactions was limited by the hierarchical dependency on the presence of the main effects, we encouraged inclusion of interaction terms by specifying a greater marginal probability of including term *X_ν _*= *X_rs_*, provided that *I_r _*= *I_s _*= 1. Specifically, we set $\left(\alpha_{1_{\mathrm{int}}},\;\alpha_{2_{\mathrm{int}}}\right)=\left(2,2\right)$ and therefore, the conditional prior probability of including term *X_rs _*was *E*(*I_rs _*= 1|*I_r _*= *I_s _*= 1) = 0.5, and the marginal prior probability was *E*(*I_rs _*= 1) = 0.03 = 0.25 × 0.25 × 0.5.

A prior for the coefficients *β_ν _*was specified via

$$\beta_{\nu }\sim \{\begin{array}{ll} N(0,{\left(\psi_{t}\sigma \right)}^{2}) & I_{\nu }=1\\ 0 & I_{\nu }=0 \end{array}.$$

Thus, if *I_ν _*= 1, the variance of *β_ν _*was the product of a fixed component *σ*^2 ^and of a component $\psi_{t}^{2}\text{, }t\in \left\{main,\;int\right\}$ depending on whether *X_ν _*was a main effect or an interaction. Specifically, we set $\psi_{main}^{2}=1$, such that *σ*^2^corresponded to the variance in main effect coefficients, and $\psi_{int}^{2}$ modeled the change in that variance for interactions terms. To allow for updating via the Gibbs sampler when no interaction term was present in the current model, a slightly informative prior was chosen for $\psi_{int}^{2}$ via log(*ψ_int_*) ~ *N*(2.3,1).

An uninformative prior distribution was placed on *σ*^2^. Since an inverse gamma prior, often chosen for the sake of its conditional conjugacy, leads to improper posterior distributions and sensitivity of inference to hyperparameter choice [7], we specified a half-Cauchy prior distribution with scale 100, as proposed by Gelman [7].

Note that via the above prior choices we a priori distinguished main and interaction effects via their model inclusion probabilities and the assumed variance of their coefficients. However, within these two groups variables were treated as exchangeable due to lack of prior evidence suggesting otherwise.

The approach was implemented using the software WinBUGS [8], running 20,000 iterations and discarding the first 2,000 as burn-in to ensure independence of the results from the initial values. Another 2,000 iterations were discarded through the default settings of WinBUGS to allow the Markov Chain to converge; hence inference was based on 16,000 iterations. We visually inspected plots of the sampled values to ensure convergence of the chain (data not shown). WinBUGS code for our analysis is provided [see Additional file 1].

## Results

### Logistic regression

Significant associations in univariate logistic regression analysis were found for packyears and *NAT1*, as indicated by OR_packyears _= 1.08, (p = 0.04, 95%-CI = 1.00-1.16) and OR*_NAT1 _*= 1.21, (p = 0.04, 95%-CI = 1.01-1.44). We also tested interactions together with their main effects and found evidence for the interaction of packyears with *NAT2 *slow acetylator status (OR_packyears × *NAT2 *_= 1.19, p = 0.02, 95%-CI = 1.03-1.38). Estimates did not change substantially when the model contained all main effects (Model_main _in Table 2), or when all pairwise interactions (Model_all_) were included. Regression with backwards selection retained packyears, *NAT1, GSTM1 *and *CYP1B1 *as explanatory variables when applied to Model_main_, and in addition the interactions of packyears with *NAT2 *and *GSTM1 *when applied to Model_all _(results not shown). For clarity, Table 2 only tabulates interactions that showed positive findings from any of the analysis approaches.

**Table 2:** Selected results from logistic regression and BMA.

Variable	Logistic regression			BMA			
	Pointwise	Model_main_^a^	Model_all_^b^				
	OR (95% CI)	OR (95% CI)	OR (95% CI)	OR (95% CI)^&^	OR|*I *= 1 (95% CI)^$^	Pr_post_	BF(*I *= 1)
**packyears**	1.08 (1.00-1.16)*	1.08 (1.01-1.16)*	1.09 (1.01-1.17)*	**1.01 (1.01-1.01)**	**1.08 (1.08-1.08)**	**0.13**	**0.43**
NAT1 *10	1.21 (1.01-1.44)*	**1.18 (0.98-1.42)^#^**	**1.21 (1.00-1.46)^#^**	**1.05 (1.05-1.05)**	**1.19 (1.19-1.20)**	**0.26**	**1.05**
*NAT2 *slow vs fast^1^	0.86 (0.70-1.04)	0.90 (0.73-1.11)	0.89 (0.72-1.09)	0.98 (0.98-0.98)	0.87 (0.87-0.88)	0.13	0.45
CYP1A1 *1^2^	0.96 (0.77-1.21)	0.94 (0.74-1.18)	0.92 (0.73-1.16)	1.00 (1.00-1.00)	0.96 (0.96-0.97)	0.06	0.19
*GSTT1 *deletion	0.84 (0.65-1.10)	0.86 (0.66-1.12)	0.86 (0.66-1.13)	0.98 (0.98-0.98)	0.86 (0.86-0.86)	0.12	0.42
*GSTM1 *deletion	1.13 (0.93-1.37)	1.15 (0.94-1.40)	1.16 (0.95-1.42)	1.01 (1.01-1.01)	1.12 (1.12-1.13)	0.09	0.31
CYP1B1 *3	0.90 (0.78-1.03)	0.90 (0.78-1.04)	0.90 (0.78-1.04)	0.99 (0.99-0.99)	0.91 (0.90-0.91)	0.09	0.31
							
**packyears × *NAT2***	1.19 (1.03-1.38)*		1.20 (1.03-1.40)*	**1.00 (1.00-1.00)**	**1.19 (1.18-1.20)**	**0.01**	**0.30**
packyears × *GSTM1*	1.11 (0.96-1.28)		1.11 (0.96-1.29)	1.00 (1.00-1.00)	1.10 (1.08-1.12)	0.00	0.07

Adjusted for age, menopause and family history

^1 ^slow acetylators had at least one *4 allele

^2 ^the reference was homozygote for the *1 allele

^a ^only main effects

^b ^all main effects and interactions

^&^OR and CI computed from mean coefficient estimate and its standard error averaged over all models

^$ ^OR and CI computed from mean coefficient estimate and its standard error averaged over all models containing the respective variable

^# ^p < 0.1, * p < 0.05

### BMA

For BMA, posterior odds ratios and 95%-confidence intervals (CI) were calculated based on the coefficients *β_ν _*across all models (marginal odds ratios) and across all models with *I_ν _*= 1 (conditional odds ratios). We also report the posterior probability that a variable is included in the model, expecting that variables harboring an association with disease will be included more frequently. Significance of findings was assessed via Bayes factors (BF), the ratio of posterior to prior odds that a variable was included in the model [9]. Thus evaluation of support of a non-zero coefficient took into account the specified prior distribution. Two model Bayes factors were computed in a similar fashion to evaluate support of a selected model versus (i) competing models, and (ii) the null model.

In our prior specifications we emphasized sparse models, resulting in coefficient estimates of 0 for many terms. Hence, expected values of marginal posterior odds ratios showed considerable shrinkage towards 1 with tight confidence intervals due to the hierarchical model (Table 2). The largest effect was observed for *NAT1 *with OR*_NAT1 _*= 1.05 (95%-CI = 1.05-1.05). To assess magnitude of effects conditional on model inclusion we also tabulate expected odds ratios and confidence intervals conditional on model inclusion. The resulting values were similar to the three logistic regression scenarios, but again with much tighter confidence intervals.

The latent indicator variable *I *was used to compute the posterior probability of model inclusion for each variable. The most frequently selected predictors were *NAT1 *(Pr_posterior _= 0.26), *NAT2 *(Pr_posterior _= 0.13) and packyears (Pr_posterior _= 0.13). The posterior probability for the interaction term of packyears and *NAT2 *was decreased at 0.01, due to the additional restriction that both main effects had to be present in the model.

We used Bayes factors (BFs), the ratio of posterior and prior odds that a variable was selected into the model, to assess the significance of a result in relation to the prior that had been assumed before the analysis. The following calibration has been proposed by Kass and Raftery [9] to interpret Bayes factors: between 1 and 3 suggests very mild evidence, between 3 and 20 positive evidence, between 20 and 150 strong, and above 150 very strong evidence for an association. Based on these guidelines, very mild evidence was found for *NAT1 *(BF = 1.05), while all other terms exhibited Bayes factors below 1.

On the model level we first computed the Bayes factor BF_all _for a specific model against all remaining models to assess whether that model was superior to the competing models. Secondly, a Bayes factor BF_0 _was computed comparing the model to the NULL model, measuring whether any additional insight was gained in relation to the model that included only age, family history and menopausal status. To facilitate interpretation, the prior and posterior odds used in the calculation of the Bayes factors are reported along with the Bayes factors in Table 3.

**Table 3:** Model results for selected models.

Model *M*	Posterior^1^	Prior^2^	BF_all_^3^	BF_0_^4^
**NULL**	**0.59**	**0.154**	**3.8**	**1.0**
packyears	0.067	0.047	1.4	0.1
***NAT1***	**0.159**	**0.047**	**3.4**	**0.3**
*NAT2*	0.063	0.047	1.3	0.1
*CYP1A1*	0.024	0.047	0.5	0.0
*GSTT1*	0.054	0.047	1.1	0.1
*GSTM1*	0.038	0.047	0.8	0.1
*CYP1B1*	0.044	0.047	0.9	0.1
*NAT1*, *GSTT1*	0.021	0.015	1.4	0.0
*NAT1*, *GSTM1*	0.017	0.015	1.1	0.0
*NAT1*, *CYP1B1*	0.017	0.015	1.1	0.0

Adjusted for age, menopause and family history

^1 ^obtained from MCMC-sampling

^2 ^uses BMA parameters

^3 ^support for *M *against all other models

^4 ^support for *M *against null model

In the comparison of one model to all remaining ones, we found positive evidence for the null model (BF_all _= 3.8) and for the model containing only *NAT1 *(BF_all _= 3.4). Very mild evidence was suggested for the single-effect models of packyears (BF_all _= 1.4), *NAT2 *(BF_all _= 1.3) and *GSTT1 *(BF_all _= 1.1). Very mild evidence was also found for the combination of *NAT1 *with each *GSTT1*, *GSTM1*, and *CYP1B1 *(BF_all _= 1.4, 1.1, and 1.1, respectively). When taking into account interactions, none of the models exhibited a Bayes factor greater than 1. The same was true for Bayes factors versus the null model.

### Sensitivity analysis

We investigated the sensitivity of our results to the choice of priors by considering different values for the prior hyperparameters described above. Specifically, we varied the values of $\left(\alpha_{1_{main}},\alpha_{2_{main}}\right)$ and $\left(\alpha_{1_{\mathrm{int}}},\alpha_{2_{\mathrm{int}}}\right)$ in the prior of *p_ν _*= Pr(*I_ν _*= 1) to consider different expected prior probabilities of model inclusion. Moreover, different specification of *μ*_int _in the prior of *ψ*_int_, as well as of the scale of the half-Cauchy prior for *σ*^2^, were evaluated. Table 4 shows the different hyperparameter choices.

**Table 4:** Hyperparameter scenarios for the sensitivity analysis.

Variation of p*_ν_*:			
**E(p_main_)**	0.10	0.25	0.50
			
**E(p_int_)**	0.25	0.50	0.75

**Variation of *ψ*_int_:**			

***μ*_int_**	2.3	3.0	4.6

**Variation of *σ*^2^:**			

**scale(*σ*^2^)**	25	100	

Estimates of posterior odds ratios showed little variation for different expected prior values of *p_ν_*. The posterior probability of model inclusion changed according to the changes in the prior parameters, i.e. doubling the prior probability of including an effect typically led to twice the posterior probability of actually including it.

Varying the mean *μ*_int _in the log-normal distribution of *ψ*_int _showed no effect on the results, neither did choosing a different scale of the prior for *σ*^2^.

## Discussion

Both logistic regression and BMA highlighted a significant effect of *NAT1*. Furthermore, logistic regression showed significant effects of packyears and of the interaction of packyears with *NAT2 *on breast cancer risk. The role of *NAT1 *as strongest effect is supported by the Bayesian analysis of selected models. Stepwise regression analysis indicated the additional involvement of *CYP1B1 *and of the interaction of packyears and *GSTM1 *in breast carcinogenesis.

On a biological level, *NAT1 *was initially implicated in breast cancer susceptibility through a report of a positive association of the NAT1*11 allele with breast cancer risk as well as combined effects with cigarette smoking and meat consumption [10], which was, however, not confirmed in a subsequent study [11]. The inconsistent results could be attributed to sample size requirements necessary for assessing effects of NAT1*11, which occurs in approximately only 3% of the general population [12]. We studied the NAT1*10 allele, which occurs with much greater frequency in the Caucasian population than the NAT1*11 allele, and may be rapid acetylating. NAT1*10 has been reported to be associated with higher NAT1 activity in both bladder and colon tissue [13-15]. However, the association between the NAT1*10 allele and increased NAT1 activity *in vivo *has not been confirmed in other studies [16-18]. For breast cancer, no significant effect of NAT1*10 has been found in several studies [10,11,19].

Detection of a gene effect with odds ratio in the order of magnitude that we have found for *NAT1 *with 80% power at a significance level of 0.05 (assuming allele frequency 0.17, population risk 10%, log-additive disease model and unmatched 1:2 case-control design) requires 1,088 cases and twice the number of controls [20]. Thus the previous studies, as well as our own, would not have enough power to consistently detect such an effect.

Our results from logistic regression analysis regarding the association of *NAT2 *with breast cancer risk, as previously reported [4], are in line with findings from other studies. In a meta- and pooled analysis including 13 studies, *NAT2 *was not independently associated with breast cancer risk but smoking was found to be associated with increased risk in *NAT2 *slow acetylators but not in rapid acetylators [21].

The *GSTM1 *null genotype has not been found to confer susceptibility to breast cancer [22]. However, smokers carrying the *GSTM1 *null genotype were at significantly elevated risk for breast cancer overall in a meta-analysis of seven studies [23]. An earlier pooled analysis of another seven smaller almost non-overlapping studies, however, did not show clear effect modification in the association between *GSTM1 *and smoking [22]. Our results from stepwise regression showed a non-significant effect modification by *GSTM1*, with higher risk of breast cancer associated with smoking among those with the *GSTM1 *null genotype.

Results from regression and Bayesian analyses differed in that univariate BMA analysis identified only *NAT1 *as significant and did not yield significant findings for packyears and the interaction of packyears and *NAT2*. One possible explanation is that inference from BMA is based on posterior and prior probabilities instead of p-values. Thereby it avoids the problem of multiple comparisons inherent in pointwise testing of coefficients in a logistic model. In fact, none of the findings from logistic regression remain significant when Bonferroni-corrected for multiple testing. However, there is no simple one-to-one correspondence between frequentist and Bayesian analyses, since the latter explicitly depend on the specified priors. Our results were stable for different hyperparameter choices. However, adequate prior specification always needs to be kept in mind before starting any Bayesian analysis. In our case, mostly uninformative prior distributions were specified to reflect the lack of sufficient external information justifying an a priori distinction of variables. We therefore allowed the data more weight versus prior information in estimation and model selection. However, if desired, BMA provides a framework for the explicit inclusion of biological prior information, like pathway characteristics, into the analysis through prior specification. If one is confident about biological prior information, stronger prior assumptions may be helpful to guide the analysis. However, bias will be introduced at the same time, so that this trade-off must be carefully considered.

## Conclusions

The strength of BMA is its explicit statement of the prior assumptions given by the prior distributions for model parameters, and its consideration of model uncertainty by obtaining results averaged over a multitude of possible models. It evaluates single variables and a range of models at the same time, yielding stabilized estimates based on a set of potential data-generating models. Moreover, it provides a means to reduce dimensionality and avoids the problem of multiple comparisons.

In our study both BMA and regression analyses yielded a significant effect of NAT1*10, while BMA attenuated other significant findings from logistic regression. Since all Bayesian inference depends on the specified prior information, prior choice must be carefully considered when conducting a Bayesian analysis.

## Competing interests

The authors declare that they have no competing interests.

## Authors' contributions

NS performed the statistical analyses and drafted the manuscript. LB participated in the statistical analyses and helped draft the manuscript. JCC conceived of the study, and participated in its design and coordination and helped draft the manuscript. All authors read and approved the final manuscript.

## Supplementary Material

Additional file 1 **WinBUGS code for the Bayes Model Averaging analysis**. The WinBUGS code for the Bayes Model Averaging analysis of our data, along with all used parameters. Click here for file

## References

[B1] Conti DV Cortessis V Molitor J Thomas DC Bayesian modeling of complex metabolic pathways Hum Hered 2003 56 83 93 10.1159/000073736 14614242

[B2] Greenland S Bayesian perspectives for epidemiological research. II. Regression analysis Int J Epidemiol 2007 36 195 202 10.1093/ije/dyl289 17329317

[B3] Chang-Claude J Kropp S Jager B Bartsch H Risch A Differential effect of NAT2 on the association between active and passive smoke exposure and breast cancer risk Cancer Epidemiol Biomarkers Prev 2002 11 698 704 12163321

[B4] Kropp S The Association between Tobacco Exposure and Breast Cancer Risk considering potentially modifying Effects of specific Genotypes PhD Thesis 2001 University of Heidelberg

[B5] Kropp S Chang-Claude J Active and passive smoking and risk of breast cancer by age 50 years among German women Am J Epidemiol 2002 156 616 626 10.1093/aje/kwf093 12244030

[B6] Hastie TJ Pregibon D Generalized linear models 1992

[B7] Gelman A Prior distributions for variance parameters in hierarchical models Bayesian Analysis 2006 1 515 533 10.1214/06-BA117A

[B8] Spiegelhalter DJ WinBUGS version 2.10 2005

[B9] Raftery AE Bayes Factors J Am Statist Assoc 1995 90 773 795 10.2307/2291091

[B10] Zheng W Deitz AC Campbell DR Wen WQ Cerhan JR Sellers TA Folsom AR Hein DW N-acetyltransferase 1 genetic polymorphism, cigarette smoking, well-done meat intake, and breast cancer risk Cancer Epidemiol Biomarkers Prev 1999 8 233 239 10090301

[B11] Millikan RC NAT1*10 and NAT1*11 polymorphisms and breast cancer risk Cancer Epidemiol Biomarkers Prev 2000 9 217 219 10698485

[B12] Loktionov A Moore W Spencer SP Vorster H Nell T O'Neill IK Bingham SA Cummings JH Differences in N-acetylation genotypes between Caucasians and Black South Africans: implications for cancer prevention Cancer Detect Prev 2002 26 15 22 10.1016/S0361-090X(02)00010-7 12088198

[B13] Hughes NC Janezic SA McQueen KL Jewett MA Castranio T Bell DA Grant DM Identification and characterization of variant alleles of human acetyltransferase NAT1 with defective function using p-aminosalicylate as an in-vivo and in-vitro probe Pharmacogenetics 1998 8 55 66 10.1097/00008571-199802000-00008 9511182

[B14] Payton MA Sim E Genotyping human arylamine N-acetyltransferase type 1 (NAT1): the identification of two novel allelic variants Biochem Pharmacol 1998 55 361 366 10.1016/S0006-2952(97)00478-4 9484803

[B15] Bruhn C Brockmoller J Cascorbi I Roots I Borchert HH Correlation between genotype and phenotype of the human arylamine N-acetyltransferase type 1 (NAT1) Biochem Pharmacol 1999 58 1759 1764 10.1016/S0006-2952(99)00269-5 10571250

[B16] Yang M Katoh T Delongchamp R Ozawa S Kohshi K Kawamoto T Relationship between NAT1 genotype and phenotype in a Japanese population Pharmacogenetics 2000 10 225 232 10.1097/00008571-200004000-00003 10803678

[B17] Badawi AF Hirvonen A Bell DA Lang NP Kadlubar FF Role of aromatic amine acetyltransferases, NAT1 and NAT2, in carcinogen-DNA adduct formation in the human urinary bladder Cancer Res 1995 55 5230 5237 7585581

[B18] Bell DA Stephens EA Castranio T Umbach DM Watson M Deakin M Elder J Hendrickse C Duncan H Strange RC Polyadenylation polymorphism in the acetyltransferase 1 gene (NAT1) increases risk of colorectal cancer Cancer Res 1995 55 3537 3542 7627961

[B19] van der Hel OL Bueno-de-Mesquita HB van Gils CH Roest M Slothouber B Grobbee DE Peeters PH Cumulative genetic defects in carcinogen metabolism may increase breast cancer risk (The Netherlands) Cancer Causes Control 2005 16 675 681 10.1007/s10552-005-1227-0 16049806

[B20] Gauderman J Morrison J QUANTO A computer program for power and sample size calculations for genetic-epidemiology studies. (1.2.4) 2006

[B21] Ambrosone CB Kropp S Yang J Yao S Shields PG Chang-Claude J Cigarette smoking, N-acetyltransferase 2 genotypes, and breast cancer risk: pooled analysis and meta-analysis Cancer Epidemiol Biomarkers Prev 2008 17 15 26 10.1158/1055-9965.EPI-07-0598 18187392

[B22] Vogl FD Taioli E Maugard C Zheng W Pinto LF Ambrosone C Parl FF Nedelcheva-Kristensen V Rebbeck TR Brennan P Glutathione S-transferases M1, T1, and P1 and breast cancer: a pooled analysis Cancer Epidemiol Biomarkers Prev 2004 13 1473 1479 15342448

[B23] Terry PD Goodman M Is the association between cigarette smoking and breast cancer modified by genotype? A review of epidemiologic studies and meta-analysis Cancer Epidemiol Biomarkers Prev 2006 15 602 611 10.1158/1055-9965.EPI-05-0853 16614098

