# Supplementary material for: Carcinogen metabolism, cigarette smoking, and breast cancer risk: a Bayes model averaging approach
Source: Epidemiol Perspect Innov. 2010 Nov 16;7:10. doi: 10.1186/1742-5573-7-10 (PMC2999590; doi:10.1186/1742-5573-7-10)
Supplement: Additional file 1 — WinBUGS code for the Bayes Model Averaging analysis. The WinBUGS code for the Bayes Model Averaging analysis of our data, along with all used parameters. [file 1742-5573-7-10-S1.DOC]

The following data was used:

list(N = 1739, start = c(1, 11), end = c(10, 23), V = 23, L = 2, psi.mu = c(0, 2.3), psi.tau = c(1000000, 1), a1 = c(2, 3), a2 = c(2, 1), u = c(1,1,1,1,1,1,1,0,0,0,1,1,1,1,1,1,1,1,1,1,1,1,1),

w = c(0,0,0,0,0,0,0,1,1,1,0,0,0,0,0,0,0,0,0,0,0,0,0))

X was the matrix of exposures, Y the vector of case/control status, and r the matrix describing the hierarchical dependencies between main and interaction effects. The below WinBUGS code has been modified and extended from Conti DV, Cortessis V, Molitor J, Thomas DC: Bayesian modeling of complex metabolic pathways. *Hum Hered* 2003, 56:83-93.

model{

for(n in 1:N) {

Y[n] ~ dbern(mu[n])

logit(mu[n]) <- alpha + inprod(b[], X[n,])

}

gamma[V+1] <- 1

for(l in 1:L) {

psi.prec[l] <- 1/psi[l]

log(psi[l]) <- psi.eta[l]

psi.eta[l] ~ dnorm(psi.mu[l], psi.tau[l])

o[l] <- psi.prec[l] * tau.eta

for(m in start[l]:end[l]) {

rho[m] ~ dbeta(a1[l], a2[l])

g.tempraw[m] ~ dbern(rho[m])

g.temp[m] <- g.tempraw[m] * u[m] + w[m]

gamma[m] <-g.temp[m]*gamma[r[m,1]]*gamma[r[m,2]]

beta[m] <- xi * eta[m]

eta[m] ~ dnorm(0, o[l])

b[m] <- gamma[m]*beta[m]

}

}

tau.eta ~ dgamma(0.5, 0.5)

xi ~ dnorm(0, tau.xi)

tau.xi <- sqrt(1/100)

sigma <- abs(xi)/sqrt(tau.eta)

alpha ~ dnorm(0.0, 1.0E-6)

}
